# The significance of the co-existence of osteopontin and tumor-associated macrophages in gastric cancer progression

**DOI:** 10.1186/s12885-015-1114-3

**Published:** 2015-03-15

**Authors:** Chang-Ni Lin, Chih-Jung Wang, Ying-Jui Chao, Ming-Derg Lai, Yan-Shen Shan

**Affiliations:** 1 Institute of Basic Medical Sciences, College of Medicine, National Cheng Kung University, Tainan, Taiwan; 2 Department of Surgery, National Cheng Kung University Hospital, College of Medicine, National Cheng Kung University, Tainan, Taiwan; 3 Institute of Clinical Medicine, College of Medicine, National Cheng Kung University, Tainan, Taiwan

**Keywords:** Gastric cancer, Osteopontin, Tumor-associated macrophage, Biomarker, Cancer immunology

## Abstract

**Background:**

Osteopontin (OPN) can recruit macrophages to the site of inflammation and promote tumorigenesis. M2 tumor-associated macrophages (M2-TAMs) also play an important role in cancer progression. This study aimed to clarify the role of OPN and M2-TAMs co-existence in gastric cancer.

**Methods:**

The levels of OPN and M2-TAMs were evaluated by immunohistochemical staining in 170 resected gastric cancer specimens that were collected from 1998 to 2012. M2-TAMs were identified by staining for an M2 marker, CD204. The prognostic significance and correlation between OPN and CD204 expression were analyzed. A co-culture system of OPN^+^-AGS and U937 cells was designed to study the effect of OPN on the skewing of macrophages toward M2-TAMs for gastric cancer progression *in vitro* and *in vivo*.

**Results:**

Patients with high expression (>50%) of OPN or CD204 exhibited poor 5-year overall survival rates (48.61%, *p* = 0.0055, and 52.14%, *p* = 0.0498, respectively). A positive correlation was observed between OPN and CD204 expression and high co-expression of OPN and CD204 demonstrated poor 5-year overall survival rates (48.90%, *p* = 0.0131). In the co-culture study, OPN was able to attract U937 cells and skew them toward M2-TAMs through paracrine action. The M2-TAMs could increase the invasiveness of OPN^+^-AGS cells and the growth rate of xenograft of a mixture of co-cultured OPN^+^-AGS and U937 cells.

**Conclusion:**

OPN can skew macrophages toward M2-TAMs during gastric cancer progression. The co-existence of OPN and infiltrating M2-TAMs correlates with disease progression and poor survival and thus can serve as a prognostic marker in gastric cancer.

## Background

Gastric cancer is the second leading cause of cancer death worldwide with overall 5-year survival rates of less than 10% [1]. The depth of invasion, extensive lymph node metastasis, and peritoneal seeding are the main reasons for high recurrence and mortality [2]. Surgery remains the only curative therapy for gastric cancer, although some studies reported that adjuvant chemotherapy and chemoradiation therapy can improve patient outcomes of resectable gastric cancers [3-5]. More than 50% of gastric cancer patients who underwent radical resection ultimately suffered from local recurrence and distant metastasis [6]. Therefore, a comprehensive investigation of the molecular mechanisms underlying the development and progression of gastric cancer is critical for designing better therapeutic strategies for the treatment of gastric cancer.

Epidemiological studies reported that chronic inflammation predisposes cells to malignancy. Additionally, inhibition of chronic inflammation in patients with premalignant disease could reduce cancer risk and cancer recurrence [7], suggesting that chronic inflammation can generate a beneficial microenvironment for tumor progression and metastatic dissemination. Previous studies reported that gastric cancer is often accompanied by the phenomena of gastritis. Gastric adenocarcinoma was also found to frequently occur in areas of chronic inflammation [8,9]. Furthermore, gastritis appears to be closely associated with an increased risk of developing gastric cancer [10]. Therefore, chronic inflammation is believed to be an important factor in driving gastric cancer progression.

The tumor microenvironment is a complex milieu that comprises various inflammatory cells and a network of signaling molecules. Among the inflammatory cells, the aberrant infiltration and activation of macrophages is frequently observed in gastric inflammation and cancer [11]. The infiltrating macrophages, also termed tumor-associated macrophages (TAMs), are associated with poor prognosis in a variety of human cancers and play important roles in tumor development [12]. In gastric carcinomas, TAMs infiltration in tumors is associated with more malignant phenotypes, including tumor angiogenesis, depth of invasion, nodal status, and clinical stages [13,14]. Gastric cancer patients with a high level of TAMs infiltration demonstrated worse outcomes after surgery than those with a low level of TAMs infiltration [14]. However, the precise role of TAMs in gastric cancer remains unknown.

Osteopontin (OPN) is a secreted matrix glycoprotein that regulates a number of biological processes. The overexpression of OPN was observed in various human cancers and is associated with poor patient outcomes in a variety of cancers, such as breast cancer [15], lung cancer [16], liver cancer [17], gastric cancer [18], colon cancer [19], and cervical cancer [20]. In gastric cancer, OPN has been reported to promote cell growth, invasion, and metastasis, whereas knockdown of OPN attenuated these effects *in vitro* and *in vivo* [21,22]. We therefore sought to clarify the correlation between OPN and TAMs in gastric cancer and its clinical significance.

## Methods

### Immunohistochemistry

For immunohistochemistry, paraffin embedded samples of 170 gastric cancer patients who underwent potentially curative surgery between 1998 and 2012 at the National Cheng Kung University Hospital (NCKUH), Tainan, Taiwan were immunostained with an anti-human OPN antibody (1:200; ab8448; Abcam) and an anti-human CD204 antibody, a marker of M2 type tumor associated macrophage (M2-TAM) (1:200; ab53566; Abcam). After horseradish peroxidase (HRP)-conjugated IgG was added for 1 hour, the specimens were analyzed by ABC detection. The degrees of staining intensity were classified into four grades by comparison with the controls as follows: 0, negative (same as the negative control); A, weak staining (<25% of the area); B, moderate staining (≥25% but <50% of the area); and C, extensive staining (>50% of the area). Grade C was considered to represent high expression of the stained protein. This study was approved by Human Experimental and Ethics Committee of National Cheng Kung University Hospital (ER-98-017). The written informed consent for participation in the study was obtained from participants.

The xenografts samples were immunostained with an anti-mouse CD31 antibody (1:200; 550274; BD Pharmingen) and an anti-mouse α-smooth muscle actin (α-SMA) antibody (1:50; ab5694; Abcam). The secondary antibodies, including HRP-conjugated IgG and fluorophore-conjugated IgG, were selected for imaging.

### Co-culture method for studying paracrine effect

Cell lines including monocyte cell line U937, TAM primary cultured from gastric cancer specimens (TAM^cli^), gastric cancer cell line AGS with expression of OPN (OPN^+^-AGS), and AGS with knockdown of OPN by short hairpin RNA (*OPN*-shRNA AGS) were used for co-culture in this study. A Boyden chamber with a 4-nm pore size insert was used for co-culture. The U937 or TAM^cli^ were seeded inside in the insert, and OPN^+^-AGS or *OPN*-shRNA AGS were seeded in the base of the chamber. After incubation for 72 hours, the condition medium was collected for future chemoattractant experiments. A monoclonal antibody against OPN, or recombinant OPN (rOPN) was also used to observe the chemoattractant effects of OPN during incubation for 72 hours.

### Invasion assay

After the co-culture treatment, 5 × 10^5^ gastric cancer cells were moved onto 8-μm pore polycarbonate inserts containing Matrigel (354234; BD Pharmingen) and incubated at 37°C. After 24 hours, the membrane was torn off slowly, washed in PBS, and stained with Giemsa. These invasive cells were counted under microscopy and photographed.

### Animal model

Four- to 6-week-old nude mice were obtained from the National Laboratory Animal Center. The housing and experimental animal procedures were approved by the Institutional Animal Care and Use Committee of NCKU (IACUC 98219). Gastric cancer cells (1 × 10^6^) were intradermally injected into the nude mice either alone or mixed with U937 (1 × 10^6^) after a 72-hour co-culture. The xenografts were observed for 9 weeks until the mice were sacrificed and were paraffin-embedded for histological analysis.

### Statistical analysis

Univariate and multivariate analysis were used to compare the overall survival and the pathology variables. Receiver operating characteristic (ROC) curve analysis was used to determine the value demonstrating the highest accuracy in predicting patient outcomes. The prognostic assessment was performed by Kaplan-Meier survival and Cox Regression analysis to identify significance. Chi-squared tests were used to analyze the correlation between OPN/CD204 staining and the clinical pathologic features. The relationship between the two variables of OPN and CD204 was analyzed by a one-way ANOVA test in GraphPad Prism 5.0 software, and most of analysis was calculated by SPSS 17.0. Statistical significance was set at *p* < 0.05.

## Results

### OPN and CD204 were highly expressed in gastric tumor and correlated with disease progression

**Figure 1:**
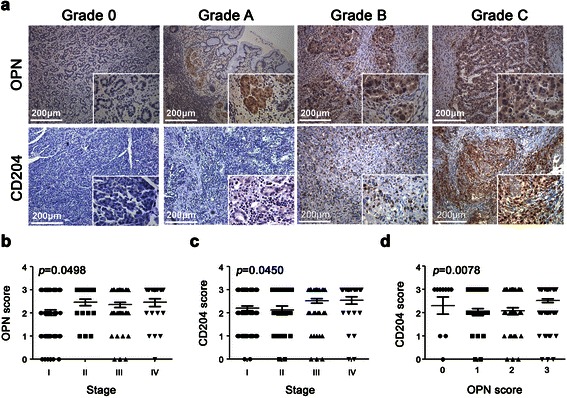
**The existence of OPN and M2-TAMs in gastric cancer specimens; the M2-TAMs were stained with a CD204-specific antibody. (a)** The stain intensity is shown from grade 0 to grade C. **(b, c)** The expression of OPN and CD204 was significantly correlated with the tumor stage (*p* < 0.05). **(d)** The OPN score was positively correlated with the CD204 score (*p* = 0.0078).

The clinicopathological characteristics of the gastric cancer patients were described in Table 1. Males constituted 57.06% (97/170) of the patient population, and 46.47% (79/170) of cases were proximal gastric cancers. Most of the cases were early gastric cancer (52.94%, 90/170). The mean follow-up time was 42.2 months (median follow-up time was 28.5 months). The different grading of OPN and CD204 expression in the gastric cancer specimens were shown in Figure 1a. A total of 53.53% of the cancer tissue samples demonstrated high expression of both OPN and CD204. The expression scores of OPN and CD204 were correlated with the tumor stage, *p* = 0.0498 and *p* = 0.0450, respectively (Figure 1b-c). Furthermore, a positive correlation was observed between OPN and CD204 expression in gastric cancer, R square (R^2^) is 0.0630 and *p* = 0.0078 (Figure 1d). The results indicated that M2-TAMs infiltration in gastric cancer tissue was correlated with OPN expression as the disease progressed.

**Table 1:** **The clinicopathological characteristics of 170 patients with gastric cancer**

	Number (N)	Percentage (%)
Gender		
Male	97	57.06
Female	73	42.94
Age		
≤50 years	34	20.00
>50 years	136	80.00
Tumor Location		
Proximal	79	46.47
Distal	91	53.53
Tumor Size (cm)		
≤5	115	67.65
>5	55	32.35
Lauren Classification		
Intestinal	73	42.94
Diffuse	97	57.06
Tumor Stage		
Early	90	52.94
Advanced	80	47.06
LN Metastasis		
No	74	43.53
Yes	96	56.47
Endpoint Status		
Survival	95	55.88
Death	75	44.12
OPN Staining		
≤50%	79	46.47
>50%	91	53.53
CD204 Staining		
≤50%	79	46.47
>50%	91	53.53

### Co-expression of OPN and CD204 was significantly associated with overall survival

**Figure 2:**
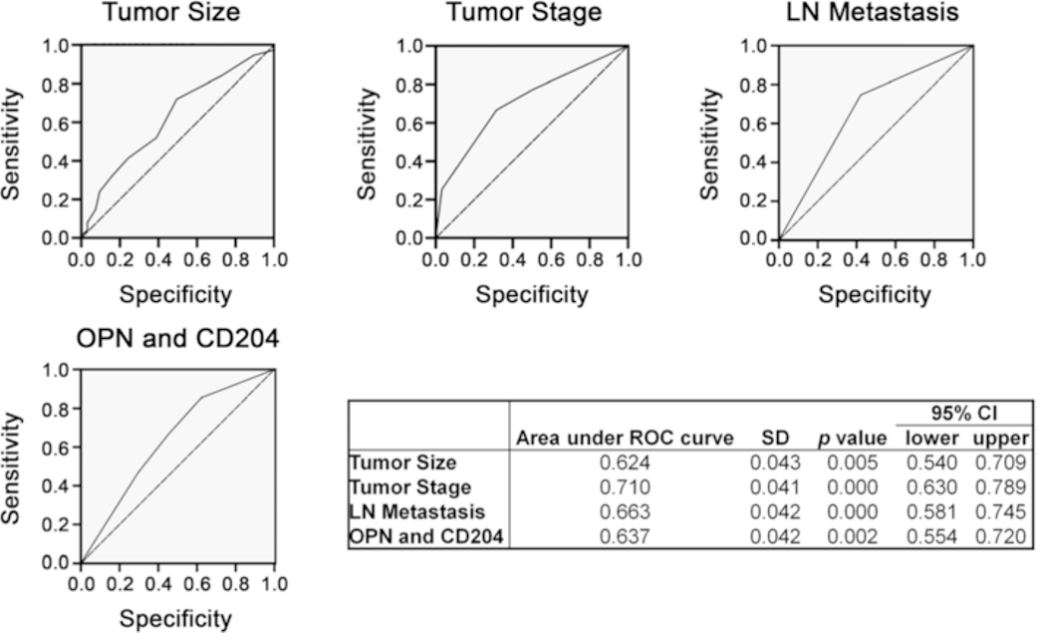
**Receiver operating characteristic (ROC) curves for the tumor size, tumor stage, lymph node metastasis, and co-expression of OPN and CD204 were used to predict overall gastric cancer survival.** The area under the ROC curve for tumor size was 0.624 (95% CI, 0.540-0.709), tumor stage was 0.710 (95% CI, 0.630-0.789), lymph node metastasis was 0.663 (95% CI, 0.581-0.745), and co-expression of OPN and CD204 was 0.637 (95% CI, 0.554-0.720). These parameters were significantly associated with overall survival (*p* = 0.005, 0.000, 0.000, and 0.002).

In univariate and multivariate analysis, the tumor size, tumor stage, lymph node metastasis, OPN expression, and CD204 expression were significantly associated with overall survival of patients with gastric cancer (Table 2). Notably, the co-expression of OPN and CD204 was more significantly associated with overall survival (*p* < 0.01) compared with OPN (*p* < 0.05) or CD204 (*p* < 0.05) alone. To confirm the correlation between the co-expression of OPN and CD204 and overall survival, ROC curve analysis was used. We found that the co-expression of OPN and CD204 was highly associated with overall survival. The tumor size (*p* = 0.005), tumor stage (*p* = 0.000), lymph node metastasis (*p* = 0.000), and co-expression of OPN and CD204 (*p* = 0.002) were significantly associated with the overall outcome (Figure 2). The results demonstrated that the co-existence of OPN and M2-TAMs in gastric cancer was highly associated with the overall survival of gastric cancer patients.

**Table 2:** **The overall survival of 170 gastric cancer patients is analyzed after resection**

	Univariate analysis (N = 170)			Multivariate analysis (N = 170)			
	Endpoint status(%)				95% CI		
	Survival	Death	*p*value	Mean	Lower	Upper	*p*value
Gender			0.317	1.425	1.349	1.501	0.320
Male	51 (53.7)	44 (46.3)					
Female	46 (61.3)	29 (38.7)					
Age			0.440	64.797	62.673	66.92	0.718
≤50 years	17 (50.0)	17 (50.0)					
>50 years	78 (57.4)	58 (43.6)					
Tumor Location			0.566	2.494	2.372	2.617	0.991
Proximal	46 (58.2)	33 (41.8)					
Distal	49 (53.8)	42 (46.2)					
Tumor Size (cm)			**0.011**	4.632	4.208	5.507	**0.006**
≤5	72 (62.6)	43 (37.4)					
>5	23 (41.8)	32 (58.2)					
Lauren Classification			0.189	2.016	1.875	2.158	0.212
Intestinal	45 (61.6)	28 (38.4)					
Diffuse	50 (51.5)	47 (48.5)					
Tumor Stage			**0.000**	1.491	1.419	1.563	**0.000**
Early	65 (68.4)	25 (33.3)					
Advance	30 (31.6)	50 (66.7)					
LN Metastasis			**0.000**	0.584	0.512	0.656	**0.000**
No	55 (74.3)	19 (25.7)					
Yes	40 (41.7)	56 (58.3)					
OPN Staining			**0.015**	2.266	2.125	2.407	**0.026**
≤50%	52 (65.8)	27 (34.2)					
>50%	43 (47.3)	48 (52.7)					
CD204 Staining			**0.034**	2.304	2.169	2.439	**0.049**
≤50%	51	28					
>50%	44	47					
OPN vs. CD204 Staining			**0.007**	2.678	2.494	2.861	**0.001**
≤50% vs. ≤50%	36 (76.6)	11 (23.4)					
≤50% vs. >50%	16 (53.3)	14 (46.7)					
>50% vs. ≤50%	15 (50.0)	15 (50.0)					
>50% vs. >50%	28 (44.4)	35 (55.6)					

Statistically significant *p* values are shown in bold.

### High co-expression of OPN and CD204 was a marker of poor prognosis

Next, we used the chi-square test to evaluate the correlation between OPN/CD204 expression and clinicopathologic characteristics (Table 3). We noted that the tumor size (*p* = 0.031), endpoint status (*p* = 0.015), and CD204 expression (*p* < 0.001) were significantly correlated with OPN expression. In the CD204 analysis results, the tumor stage (*p* = 0.006), endpoint status (*p* = 0.034), and OPN expression (*p* < 0.001) were also significantly correlated. Interestingly, OPN expression was significantly correlated with CD204 expression in gastric cancer. In conjunction with the results shown in Figure 1d, M2-TAMs infiltration in gastric tumors was highly correlated with OPN expression. Thus, we next analyzed whether co-expression of OPN and CD204 correlated with clinicopathologic characteristics. Similarly, the tumor stage (*p* = 0.004) and endpoint status (*p* = 0.007) were significantly correlated with the co-expression of OPN and CD204. The results prove that the co-expression of OPN and CD204 was associated with the tumor stage and predicted worse patient outcomes.

**Table 3:** **OPN and CD204 expression correlated with clinicopathologic characterization**

	OPN expression (%)			CD204 expression (%)			OPN vs. CD204 expression (%)				
	≤50%	>50%	*p*value	≤50%	>50%	*p*value	≤50% vs. ≤50%	≤50% vs. >50%	>50% vs. ≤50%	>50% vs. >50%	*p*value
Gender			0.550			0.981					0.795
Male	47 (59.5)	50 (54.9)		45 (57.0)	52 (57.1)		29 (61.7)	17 (56.7)	15 (50.0)	36 (57.1)	
Female	32 (40.5)	41 (45.1)		34 (43.0)	39 (42.9)		18 (38.3)	13 (43.3)	15 (50.0)	27 (42.9)	
Age			0.758			0.489					0.606
≤50 years	15 (19.0)	19 (20.9)		14 (17.7)	20 (22.0)		7 (14.9)	8 (26.7)	7 (23.3)	12 (19.0)	
>50 years	64 (81.0)	72 (79.1)		65 (82.3)	71 (78.0)		40 (85.1)	22 (73.3)	23 (76.7)	51 (81.0)	
Tumor Location			0.146			0.480					0.229
Proximal	32 (45.5)	47 (51.6)		39 (49.4)	40 (44.0)		22 (46.8)	9 (30.0)	16 (53.3)	32 (50.8)	
Distal	47 (59.5)	44 (48.4)		40 (50.6)	51 (56.0)		25 (53.2)	21 (70.0)	14 (46.7)	31 (49.2)	
Tumor Size (cm)			**0.031**			0.068					0.135
≤5	60 (75.9)	55 (24.1)		59 (74.7)	56 (61.5)		38 (80.9)	20 (66.7)	19 (63.3)	38 (60.3)	
>5	19 (60.4)	36 (39.6)		20 (25.3)	35 (38.5)		25 (19.1)	21 (33.3)	14 (36.7)	31 (39.7)	
Lauren Classification			0.519			0.738					0.983
Intestinal	36 (45.6)	37 (40.7)		35 (44.3)	38 (41.8)		21 (44.7)	13 (43.3)	12 (40.0)	27 (42.9)	
Diffuse	43 (54.4)	54 (59.3)		44 (55.7)	53 (58.2)		26 (55.3)	17 (56.7)	18 (60.0)	36 (51.1)	
Tumor Stage			0.379			**0.006**					**0.004**
Early	46 (58.2)	44 (48.4)		53 (67.1)	37 (40.7)		34 (72.3)	11 (36.7)	18 (60.0)	27 (42.9)	
Advance	33 (41.8)	47 (51.6)		26 (32.9)	54 (59.3)		13 (27.7)	19 (63.3)	12 (40.0)	36 (57.1)	
LN Metastasis			0.263			0.082					0.345
No	38 (48.1)	36 (39.6)		40 (50.6)	34 (37.4)		25 (53.2)	12 (40.0)	14 (46.7)	23 (36.5)	
Yes	41 (51.9)	55 (60.4)		39 (49.4)	57 (62.6)		22 (46.8)	18 (60.0)	16 (53.3)	40 (63.5)	
Endpoint Status			**0.015**			**0.034**					**0.007**
Survival	52 (65.8)	43 (47.3)		51 (64.6)	44 (48.4)		36 (76.6)	16 (53.3)	15 (50.0)	28 (44.4)	
Death	27 (34.2)	48 (52.7)		28 (35.4)	47 (51.6)		11 (23.4)	14 (46.7)	15 (50.0)	35 (55.6)	

Statistically significant *p* values are shown in bold; *p* values were calculated using Fisher’s exact test.

**Figure 3:**
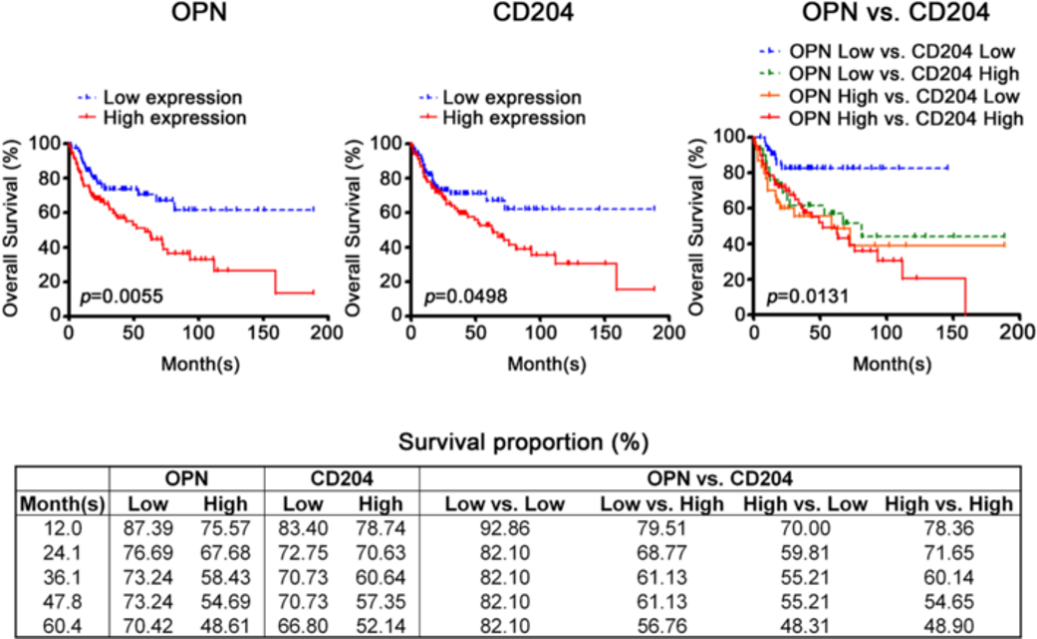
**The overall survival of gastric cancer patients with variable OPN and CD204 expression was analyzed**. Either high OPN expression (>50% positive staining, *p* = 0.0055) or high CD204 expression (>50% positive staining, *p* = 0.0498) in gastric cancer was correlated with lower overall survival. However, the most significant reduction in overall survival occurred for patients with high co-expression of OPN and CD204 (*p* = 0.0131). The 5-year survival rate in high OPN expression patients was 48.61%, in low OPN expression was 70.42%, in low CD204 expression was 66.80%, in high CD204 expression was 52.14%, in high co-expression of OPN and CD204 was 48.9%, and in low co-expression of OPN and CD204 was 82.10%.

Kaplan-Meier survival analysis was used to determine the overall survival of patients with gastric cancer (Figure 3). Patients with high expression of OPN demonstrated significantly worse overall survival than those with low expression of OPN (*p* = 0.0055) and the hazard ratio is 2.039 (95% CI of ratio is 1.220 to 3.406). The 5-year survival rate of gastric cancer patients with high expression OPN was 48.61%, whereas for patients with low expression OPN the 5-year survival rate was 70.42%. In M2-TAMs analysis, we found that patients with high expression of CD204 exhibited lower overall survival (p = 0.0498), hazard ratio is 1.653 (95% CI of ratio is 0.995 to 2.745) and a lower 5-year survival rate (52.14%), compared with low CD204 expressing patients. Furthermore, the 5-year survival rate of patients with high co-expression of OPN and CD204 was 48.90%, whereas that of patients with low co-expression of OPN and CD204 was 82.10%. These results suggest that high co-expression of OPN and CD204 was a marker of poor prognosis in gastric cancer.

### A paracrine regulation between OPN and M2-TAMs in gastric cancer

**Figure 4:**
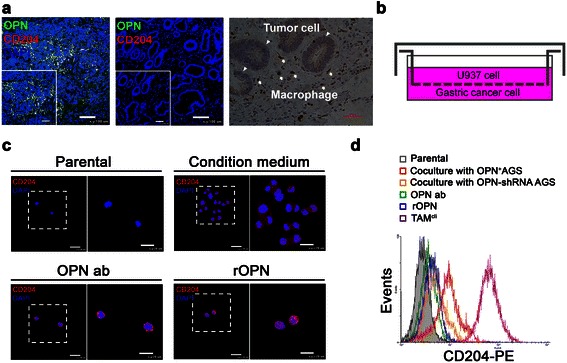
**The paracrine effect of OPN skewed macrophages toward M2-TAMs in gastric cancer. (a)** Dual immunofluorescence staining of OPN (green) and CD204 (red) shows co-localization (yellow) in gastric cancer specimens. The immunohistochemistry of CD204 shows that macrophages did not come in contact with tumor cells. **(b)** A co-culture method was designed to mimic the tumor microenvironment of gastric cancer without direct contact between cancer cells and macrophages. **(c)** The confocal images show that OPN contributed to the recruitment of U937 cells and skewed the cells toward M2-TAMs after treatment with conditioning medium; this phenomenon could be blocked by an OPN monoclonal antibody. Recombinant OPN was able to increase CD204 expression on macrophages. **(d)** The amount of CD204^+^TAMs was shown by flow cytometry. The CD204^+^-TAM phenotype increased dramatically after co-culture with OPN^+^AGS cells (red, open histogram). Parental cells (filled histogram), *OPN*-shRNA AGS cells (orange, open histogram), OPN neutralizing antibody (green, open histogram), or recombinant OPN (blue, open histogram) were compared. The TAM^cli^ cells isolated from human gastric cancer were used as a positive control.

The dual immunofluorescence results indicated that OPN and CD204 were co-localized even in high-expression or low-expression gastric cancer specimens. This finding suggests that OPN was bound to the surface of M2-TAMs. The IHC staining demonstrated that the macrophages were located beside the tumor cells (Figure 4a). The results implied that paracrine regulation occurs between OPN and M2-TAMs within gastric cancer. Thus, we designed a co-culture system to mimic the tumor microenvironment. To explore the chemoattractant effect of OPN on M2-TAMs infiltration, U937 (5 × 10^5^) were cultured in the insert, and OPN^+^-AGS gastric cancer cells were grown on the base of a Boyden chamber (Figure 4b). After incubated for 72 hours, the condition medium was collected and added into another lower chamber. New insert containing U937 cells was put into the chamber and then incubated for 72 hours. The number of M2-TAMs in the lower chamber was significantly increased, compared with those cells treated with conditioning medium containing a monoclonal OPN antibody (Figure 4c). Furthermore, when rOPN was added to the normal medium, M2-TAMs were still observed in the base of chamber. Flow cytometry further proved that the M2-TAMs differentiation was associated with the presence of OPN (Figure 4d). Taken together, these results suggest that OPN was required for M2-TAMs infiltration in gastric cancer.

**Figure 5:**
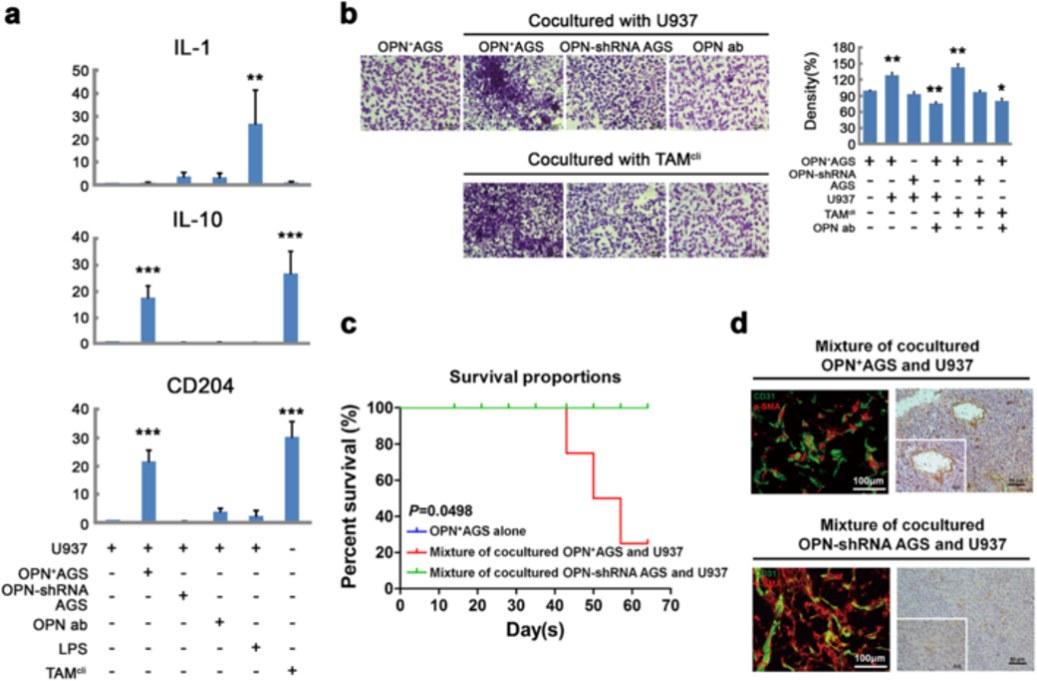
**The co-expression of OPN and M2-TAMs promotes gastric tumorigenesis. (a)** The expression of M1/M2 markers in U937 cells was analyzed after co-culture treatment by real-time PCR. LPS-treated U937 cells expressed IL-1 as M1 macrophages, and U937 cells alone were used as a negative control. TAM^cli^ cells isolated from clinical specimens were used as a positive control. After co-culture with OPN^+^-AGS cells, the U937 cells differentiated into M2-TAMs expressing high levels of CD204 and IL-10. **(b)** After 72 hours of co-culture with U937 or TAM^cli^ cells, the invasiveness of the OPN^+^-AGS cells increased but that of *OPN*-shRNA AGS cells did not. The increased cell invasiveness was also blocked by an OPN monoclonal antibody. **(c)** The mixture of co-cultured OPN^+^-AGS and U937 cells inoculated into nude mice showed poor survival compared with a mixture of co-cultured *OPN*-shRNA AGS and U937 cells or OPN^+^-AGS cells alone (*p* = 0.0498). **(d)** The neovascularization in the xenografts was shown by double-staining with anti-CD31 and anti-αSMA antibodies. Less neovascularization with normal vascular structure was found in xenografts of a mixture of co-cultured *OPN*-shRNA AGS and U937 cells.

After being co-cultured with OPN^+^-AGS cells, the U937 cell demonstrated significantly increased mRNA levels of CD204 and IL-10, as in TAM^cli^ cells, when compared with cells co-cultured with *OPN*-shRNA AGS cells, stimulated with LPS (to become M1 macrophages exhibiting increased mRNA levels of IL-1) or treated with an OPN monoclonal antibody (Figure 5a). This result proved that OPN was able to recruit monocytes and skewed them toward a M2-TAMs phenotype in gastric cancer microenvironments.

### M2-TAM promoted gastric cancer progression

After being co-cultured with U937 or TAM^cli^ cells for 3 days, the invasiveness of OPN^+^-AGS cells was significantly increased, but the invasiveness was reduced after the addition of OPN antibodies (Figure 5b). To assess the effects of TAMs on tumorigenesis *in vivo*, a mixture of co-cultured OPN^+^-AGS and U937 cells was inoculated into the back skin of nude mice to observe the growth of xenografts. Compared with inoculation of OPN^+^-AGS cells alone, the xenografts from mixture of co-cultured OPN^+^-AGS and U937 cells grew faster and disseminated to the liver and peritoneal cavity, similar to human gastric cancer. Those nude mice exhibited poor survival compared with mice inoculated with a mixture of *OPN*-shRNA and U937 cells or AGS cells alone (Figure 5c). We also found that a marked neovascularization occurred in the xenografts from a mixture of co-cultured OPN^+^-AGS and U937 cells, but the neovascularization was reduced in the tumors generated from a mixture of *OPN*-shRNA AGS and U937 cells (Figure 5d). Collectively, these results proved that OPN could recruit macrophages and skew them toward M2-TAMs formation and the M2-TAMs further promoted gastric cancer progression.

## Discussion

Recently, many studies have proven that the infiltrating inflammatory cells in the tumor microenvironment could promote cancer progression [23,24]. Chronic inflammation is frequently found within gastric tumors, and M2-TAMs can be observed after staining for M2 markers. The correlation between the presence of M2-TAMs in tumors and poor survival has been demonstrated in several cancer types [25-27]. However, the reasons underlying the ability of infiltrating TAMs to promote cancer progression remain a mystery. We observed that OPN was highly expressed in gastric cancer specimens and positively correlated with M2-TAMs infiltration (R^2^ = 0.7743). More importantly, the co-existence of OPN and M2-TAMs is significantly correlated with poor prognosis and lower 5-year survival rates. Although our results are similar to those of previous reports, we focused on M2-type macrophages (CD204 staining) rather than total macrophages (CD68 staining) [28,29]. In addition, a previous study demonstrated that OPN overexpression in TAMs was able to enhance angiogenesis and growth in melanoma through autocrine signaling [30] rather than through the interactions with tumor cells. Therefore, this is the first study to prove the paracrine regulation of M2-TAMs by OPN to promote gastric cancer progression.

OPN is a secreted glycoprotein that can generate macrophage accumulation [31] and enhance tumor invasion [32,33]. However, the detailed mechanism remains unclear. In this study, we used a co-culture system to demonstrate a paracrine regulation between OPN and M2-TAMs in gastric cancer. OPN has the ability to recruit and skew macrophages toward M2-TAMs and thus promotes gastric cancer progression. OPN has been reported to promote invasion and metastasis of gastric cancer through HIF-1α upregulation and MMP9 activation [34]. Moreover, the plasma OPN concentration in patients with metastatic disease is significantly higher than that in patients without metastases [35]. In breast cancer, OPN can promote cancer progression, whereas knockdown of OPN aborts this effect [36]. Notably, contradictory results were observed in an *OPN* knockout squamous carcinoma mouse model. Primary skin tumors grew larger and produced more numerous lung metastases in *OPN*-deficient mice, compared with their wild-type counterparts [37]. The controversial findings may result from the different functions of OPN in normal tissues and tumors. Our results are consistent with a previous study which reported that the tumor microenvironment determines the effects of OPN [38]. In the future, we will further clarify the mechanism underlying the interaction between OPN and TAMs in gastric cancer.

## Conclusion

Our study clearly demonstrates that clinical parameters, including tumor size, tumor stage, lymph node metastasis, OPN expression, and TAMs infiltration are associated with overall survival of gastric cancer patients. Patients with high co-expression of OPN and CD204 exhibit a lower 5-year survival rate. *In vitro* and *in vivo* experiments further verify the interaction between OPN and TAMs, which can promote gastric cancer progression. Our novel findings provide a good marker for predicting the outcomes of patients with gastric cancer.

## Abbreviations

TAM: Tumor associated macrophage
M2-TAM: M2 type tumor associated macrophage
OPN: Osteopontin
α-SMA: α-smooth muscle actin
HRP: Horseradish peroxidase
IgG: Immunoglobulin G
shRNA: Short hairpin RNA
ROC: Receiver operating characteristic
NCKUH: National Cheng Kung University Hospital
IACUC: Institutional Animal Care and Use Committee
rOPN: Recombinant OPN
TAM^cli^: Human TAM from gastric cancer specimens
OPN^+^-AGS: Gastric cancer cell line AGS with expression of OPN
*OPN*-shRNA AGS: AGS with knockdown of OPN by short hairpin RNA
